# Investigation of *in vitro* effects of ethephon and chlorpyrifos, either alone or in combination, on rat intestinal muscle contraction

**DOI:** 10.2478/v10102-010-0002-6

**Published:** 2010-03-29

**Authors:** Mustafa Alp Çetinkaya, Emine Baydan

**Affiliations:** 1 National Food Reference Laboratory, Turkish Ministry of Agriculture and Rural Affairs, Fatih Sultan Mehmet Bulvari, Yenimahalle, Ankara, Turkey; 2 Department of Pharmacology and Toxicology, Faculty of Veterinary Medicine, University of Ankara, Ankara, Diskapi, Turkey

**Keywords:** ethephon, chlorpyrifos, acetylcholine, combination, organophosphate

## Abstract

A range of pesticides is widely used in pest management and the chances of exposure to multiple organophosphorus (OP) compounds simultaneously are high, especially from dietary and other sources. Although health hazards of individual OP insecticides have been relatively well characterized, there is lesser information on the interactive toxicity of multiple OP insecticides. The aim of this study is to elicit the possible interactions in case combined exposure of an OP pesticide chlorpyrifos (CPF) and a plant growth regulator ethephon (ETF) which are used worldwide. The ileum segments of 3 months old Wistar Albino male rats were used in isolated organ bath containing Tyrode solution. ETF and CPF were incubated (10^−7^ M concentration) separately or in combination with each other to ileum and their effects on acetylcholine-induced contractions were studied. The data obtained from this study show that, single and combined exposure to the agents caused agonistic interactions with regard to potency of ACh whereas they caused a decrease on E_max_ value of ACh. These findings suggest that exposure to these agents which have direct and indirect cholinergic effects, may cause developing clinical responses with small doses and earlier but the extent of toxicity will be lower.

## Introduction

Organophosphorus (OP) pesticides are the major chemical class of insecticides used in the world today. The primary mode of action for OP pesticides is initiated through irreversible inhibition of acetylcholinesterase (AChE), the enzyme responsible for degrading the neurotransmitter acetylcholine (Howard and Pope, 2002; Kousba *et al*., 2003).

Ethephon [(2-chloroethyl) phosphonic acid] (ETF) is a major plant growth regulator (PGR) that promotes fruit ripening, abscission, flower induction, and other responses by releasing ethylene gas, a natural plant hormone (Segall *et al*., 1991; EPA, 1995; Tomlin, 1997; Haux *et al*., 2000) which spontaneously decomposes at physiological pH (Zhang and Casida, 2002). ETF as an OP, even though it gives no cholinergic signs of poisoning, was tested the same way as an OP insecticide. It markedly inhibited plasma butyrylcholinesterase (BChE) with much less effect on brain or erythrocyte AChE in rats and mice both in vitro and in vivo (Haux *et al*., 2002).

Chlorpyrifos [O,O-diethyl O-(3,5,6-trichloro-2-pyridinyl)-phosphorothioate] (CPF), is a broad-spectrum, chlorinated organophosphate insecticide (Smegal, 2000) known to cause acute toxicity through irreversible inhibition of AChE (Chanda *et al*., 1997) via one of its major metabolites, chlorpyrifos oxon (CPO) (Cook and Shenoy, 2003). The parent OPs, like chlorpyrifos, are weak inhibitors of AChE which must first undergo CYP-mediated oxidative desulfuration to CPO, to become an inhibitor of cholinesterase activity (Cochran, 2002; Poet *et al*., 2003; El-Masri *et al*., 2004).

Oral absorption of a chemical or drug can be altered by both intestinal and liver metabolism. Although the blood flow and tissue volume of the intestine is slightly lower than the liver, the extensive microvilli structure results in a large surface area, ideally suited for absorption (Poet *et al*., 2003). The activation of CPF to CPO is mediated by cytochrome P450 mixed-function oxidases (CYP450), primarily within the liver. However, extrahepatic metabolism has been reported in other tissues (Timchalk *et al*., 2002). Enterocytes within the small intestine also possess CYP metabolic capacity, and in the case of some enzymes (i.e., 3A4) the concentration within the enterocytes is comparable to the liver (Poet *et al*., 2003). Intestinal CYP450 metabolism of drugs can be substantial, and is also likely to be an important contributor to CPF metabolism (Timchalk *et al*., 2002).

Acetylcholine is a well-recognized neurotransmitter in the gastrointestinal tract, and the activation of muscarinic cholinoceptors by acetylcholine is important in eliciting contraction of intestine and colon (Tezuka *et al*., 2004). In small bowel, acetylcholine (ACh) released from intramural cholinergic nerves acts at nicotinic cholinergic receptors to cause excitation of enteric nerves and at muscarinic cholinergic receptors to cause contractions of intestinal smooth muscle (Taha *et al*., 2002).

Organophosphate (OP) pesticides are often used in combination with one another and with the components of formulations. Exposure to multiple OP-containing pesticide formulations may lead to synergistic neurotoxicity by a direct mechanism at the cellular level (Axelrad *et al*., 2002). As a range of insecticides is extensively used in pest management, the chances of exposure to multiple OP compounds simultaneously are high, especially among agricultural and public health workers. Furthermore, from dietary and other sources, there may be separate but closely timed exposures to such insecticides. Although health hazards of individual OP insecticides have been relatively well characterized, there is lesser information on the interactive toxicity of multiple OP insecticides (Karanth *et al*., 2004).

The chance of exposing multiple pesticides from different sources, especially by ingestion of different kind of food, is very high, therefore the aim of current study is to investigate possible interactions of two different kind of widely used pesticides (a plant growth regulator ethephon and an insecticide chlorpyrifos) either alone or in combination, on ACh-induced contractions on rat ileal smooth muscle.

## Materials and methods

### Animals and Tissue Preparation

Three months old male Wistar albino rats weighing 150–180 g were used for the experiments. Rats were fasted overnight with free access to water. Prior to the beginning of the experiments, rats were anesthetized by ether. Subsequently, the animals were sacrificed by cervical dislocation and small intestine was removed rapidly. The organ was transferred to a dish containing Tyrode solution. The study was approved by the Ethical Committee of University of Ankara, Faculty of Veterinary Medicine.

After the rinsing of the lumen and clearing of the adventitial tissue, longitudinal segments (10 mm long) were cut from ileum. The segment was tied at both ends with cotton-line, suspended under a load of 1 g in an organ bath containing 10 ml of Tyrode solution kept at 37 °C and bubbled with a mixture of 95% O_2_ and 5% CO_2_ (pH 7.4). The Tyrode solution had the following composition (mM): NaCl 136.9, KCl 2.68, CaCl_2_ 1.8, MgCl_2_.6H_2_O 1.05, NaH_2_PO_4_.2H_2_O 0.4, NaHCO_3_ 11.9 and glucose 5.5. Mechanical activity of the segments, measured as changes in isometric tension, was recorded via an isometric force transducer (FDT-10A MAY) on a MAY Polygraph model TDA99 (MAY, Ankara, TURKEY). Each preparation was allowed to equilibrate for at least 60 min prior to initiating the experimental procedure. During the stabilization period the bath solution was changed every 15 minutes.

The pD_2_ is defined as the negative logarithm of the EC_50_. The potency of a drug is commonly quantified as the EC_50_. It is the concentration of agonist required to provoke a response halfway between the baseline and maximum response (E_max_) of a dose-response curve. The value of the highest contractile response (maximal contraction) was accepted as 100% and the percentages of the other contractile responses of the concentrations were calculated. With these calculations, pD_2_ and E_max_ values were obtained by using “GraphPad Prism ver.4 for Windows”. Evaluation of the obtained responses was made by comparing the effects of agents on pD_2_ and E_max_ values of ACh.

### Assays

#### Evaluating the effects of the agents on ACh induced contractions

The effects of agents on ACh were elicited by studying a control procedure. In the first stage, with this purpose, control ACh was applied cumulatively (10^−9^–10^−4^ M semi-logarithmically) to ileum tissue and corresponding concentration-response curve was obtained. From the curve, E_max_ and pD_2_ values and the mg values of highest contractile responses were calculated. In the second stage, this was followed by single or combined incubation of agents for 10 minutes before the addition of further cumulative concentrations of ACh. Thus, single and combined effects of agents on E_max_ and pD_2_ values of ACh were observed. Between two stages, incubation medium was changed three times in 45 minutes to restore the baseline.

#### Evaluating the single incubation of the agents

ACh was applied cumulatively to ileum tissue. After obtaining the control curve, the incubation medium was changed three times in 45 minutes to restore the baseline. This was followed by incubation of single agent (ETF or CPF) for 10 minutes with 10^−7^ M concentration before the addition of further cumulative concentrations of ACh.

#### Evaluating the combined incubation of the agents

Control curve was obtained by applying ACh (10^−9^–10^−4^ M semi-logarithmically) cumulatively to ileum tissue. The incubation medium was changed three times. After restoring the base line, combined ETF (10^−7^ M) and CPF (10^−7^ M) were exposed to ileum tissue at the same time (the half of single exposure dose used for each agent) for 10 minutes and then further cumulative concentrations of ACh was added.

#### Evaluating cumulative exposure of agents on ileum tissue

The cumulative (10^−10^–10^−6^ M logarithmically) application of agents (ETF or CPF) was also studied separately to obtain the corresponding concentration-response curve. The mg values of every contractile response were calculated. The highest contractile response was accepted 100% and according to this highest response, the percentages of responses of other contractions were also calculated.

### Chemicals

Acetylcholine chloride (CAS No: 60-31-1, Sigma A6625) was purchased from Sigma Chemical Company. Ethephon (CAS No: 16672-87-0) is freely soluble in water. EFHUN 250 cc (480 g/l Ethephon), a commercial water-based product of ethephon was purchased from Agrobest Company (Ankara, TURKEY). All drugs were dissolved in distilled water. Chlorpyrifos (CAS No: 2921-88-2) was of analytical grade and purchased from Dow AgroSciences. Chlorpyrifos is practically insoluble in water, therefore, it was first dissolved in acetone (35 mg/10 ml) and then dilutions were made with distilled water.

### Statistics and data analysis

Statistical calculations and graphs are made with “GraphPad Prism ver.4 for Windows”. The data are expressed as mean ± SE. Results are expressed as a percentage of the maximum contraction of the first acetylcholine curve. All results are expressed as mean ± SEM of six or more preparations obtained from different animals. Wilcoxon Signed Ranks Nonparametric tests were used to compare ACh control curve and ACh curve after incubation of agents. The concentration needed to produce 50% contraction effect (EC_50_) was obtained from the regression plot and mean EC_50_ ± 95% confidence intervals was calculated for each dose assessed and maximum values were obtained from each concentration-response curve (E_max_). Differences were considered significant when *p*-value is <0.05.

## Results

### The effects of incubation on ACh induced contractions

#### Ethephon

The changes on pD_2_ and E_max_ values of ACh on rat ileum after incubating (10 min) with Ethephon (10^−7^ M), are shown in Table 1. According to the obtained results, the pD_2_ value of ACh increased (ACh: 6.241 ± 0.165 and ETF incubation: 6.447 ± 0.160) and the E_max_ values of ACh decreased (ACh: 94.88 ± 2.34 and ETF incubation: 82.60 ± 4.15). These changes are statistically significant (*p<*0.05).

**Table 1:** Effects of agents either alone or in combination on pD_2_ and E_max_ values of ACh on rat ileum before and after incubation.

	pD_2_	E_max_
**ACh (n = 9)**	6.241 ± 0.165	94.88 ± 2.34
**ETF (10**^**−7**^** M) incubation**	6.447 ± 0.160^*^	82.60 ± 4.15^*^
**ACh (n = 15)**	6.210 ± 0.070	94.58 ± 2.48
**CPF (10**^**−7**^** M) incubation**	6.575 ± 0.236^*^	86.26 ± 4.36^*^
**ACh (n = 19)**	6.164 ± 0.065	93.76 ± 1.52
**ETF+CPF (10**^**−7**^** M) incubation**	6.247 ± 0.069^*^	86.25 ± 3.67^*^

Data are reported as mean ± SE;

^*^ *p < *0.05 compared to control.

#### Chlorpyrifos

The changes on pD_2_ and E_max_ values of ACh on rat ileum after incubating (10 min) with Chlorpyrifos (10^−7^ M), are shown in Table 1. According to the results, the pD_2_ value of ACh increased (ACh: 6.210 ± 0.070 and CPF incubation: 6.575 ± 0.236) and the E_max_ values of ACh decreased (ACh: 94.58 ± 2.48 and CPF incubation: 86.26 ± 4.36). These changes are statistically significant (*p<*0.05).

#### Combination

The changes on pD_2_ and E_max_ values of ACh on rat ileum after incubating (10 min) simultaneously with Ethephon(10^−7^ M) and Chlorpyrifos (10^−7^ M) are shown in Table 1. Results show that the pD_2_ value of ACh increased (ACh: 6.164 ± 0.065 and [ETF+CPF] incubation: 6.247 ± 0.069) and the E_max_ values of ACh decreased (ACh: 93.76 ± 1.52 and [ETF+CPF] incubation: 86.25 ± 3.67). These changes are statistically significant (*p<*0.05).

### The effects of cumulative exposure of the agents on ileum

**Figure 1:**
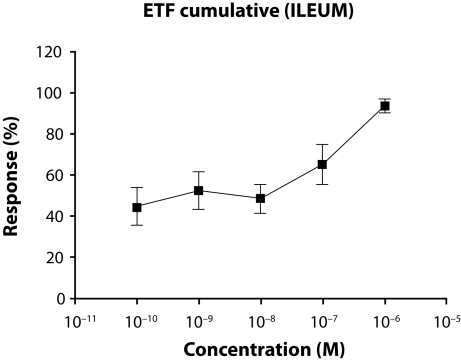
The average mg values of the highest contractions and the responses of cumulatively applied ETF on ileum.

The average mg values of the highest contractions and the responses of cumulatively applied ETF (10^−10^–10^−6^ M) on ileum are shown in Figure 1 and Table 2 respectively.

**Table 2:** The average mg values of highest contractions of cumulatively applied ETF or CPF on rat ileum.

	Highest contraction (mg ± SEM)	n
**ETF (10**^**−10**^**–10**^**−6**^** M)**	413 ± 63	10
**CPF (10**^**−10**^**–10**^**−6**^** M)**	461 ± 76	9

**Figure 2:**
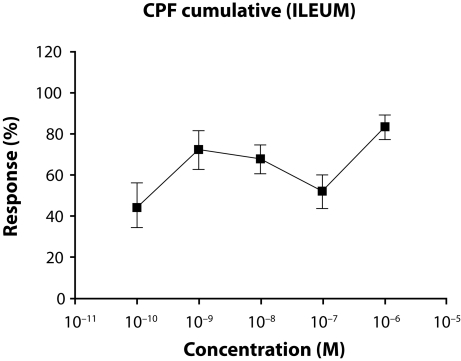
The average mg values of the highest contractions and the responses of cumulatively applied CPF on ileum.

The average mg values of the highest contractions and the responses of cumulatively applied CPF (10^−10^−10^−6^ M) on ileum are shown in Figure 2 and Table 2 respectively.

**Figure 3:**
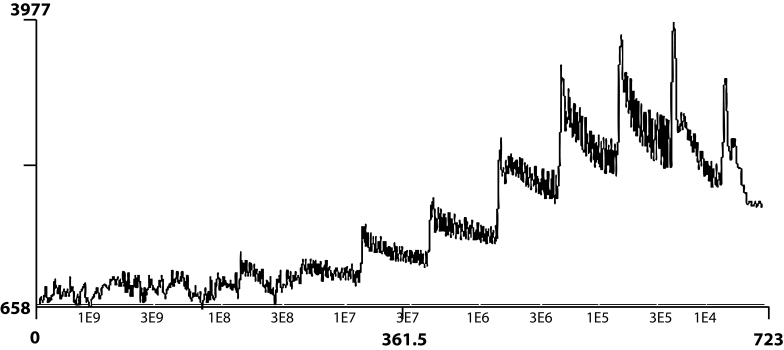
Cumulative concentration-response of ACh that is appropriate to specific graded concentration-response.

It is seen that the cumulative (10^−10^–10^−6^ M) application of ETF or CPF on rat ileum cause contractions which are not similar to specific graded concentration-response. These contractions have no graded increase related to concentrations, so appropriate E_max_ and pD_2_ values can not be calculated. Cumulative concentration-response of ACh (10^−9^–10^−4^ M, semi-logaritmically) that is appropriate to specific graded concentration-response, is shown in Figure 3.

## Discussion

There is an increase of potency of ACh and a decrease of maximum effect of ACh on rat ileum after incubating both with ETF (10^−7^ M) and with CPF (10^−7^ M) (Table 1). These results show that the ACh concentration is increased in synaptic cleft by AChE inhibition because agents act as acetylcholinesterase inhibitors and also the contractions are decreased because agents bind directly to the receptors. The changes are statistically significant but also biologically important because toxicity and desensitisation by ETF or CPF occurs earlier with lower ACh concentrations.

Similar studies which involve increase in potency of choline esters are available. According to Sparks *et al*. (1999), pretreatment of mice i.p. with ETF decreases the LD_50_ of succinylcholine in comparison with controls, that is, a toxicity increase of fourfold. Mice are sensitized to succinylcholine toxicity when their serum BuChE activity is inhibited 77–94% by ethephon with pretreatment time of 1 hour.

The maximum effect of agents (Table 2) is lower than that of ACh, therefore after incubating with ETF (10^−7^ M) or CPF (10^−7^ M), occupation of receptors by ETF or CPF may cause a decrease in E_max_ value of ACh. The down-regulation is likely responsible for this decrease. Cochran (2002) stated that adaptive responses to single and multiple doses of chlorpyrifos include down-regulation of acetylcholine receptors and reduced synthesis of acetylcholine, so that the presynaptic release of acetylcholine is restricted.

The interaction of some cholinesterase inhibitors with cholinergic receptors is known for many years. A number of studies have evaluated effects of anticholinesterases on muscarinic receptor binding. Paraoxon, malaoxon and chlorpyrifos oxon inhibited [3H]*cis*dioxolane binding in a concentration dependent manner at sub-micromolar concentrations (potencies: chlorpyrifos oxon > paraoxon > malaoxon). Chlorpyrifos oxon, paraoxon and methyl paraoxon displaced [3H]oxotremorine-M binding to rat cardiac membrane muscarinic receptors with chlorpyrifos oxon being most potent (IC_50_=7 nM) (Pope *et al*., 2005).

Similar to the responses of single exposures, combined exposure of ETF and CPF on rat ileum caused increase in pD_2_ values (*p<*0.05) and decrease in E_max_ values (*p<*0.05) of concentration-response curves of ACh (Table 1).

A number of studies have compared the single and combined exposures. These studies indicate that combinations may change potency of each other as agonistic or antagonistic manner. El-Masri *et al*. (2004) used the overall model to investigate the mechanism of interaction and to calculate interaction threshold doses at various dose levels of CPF and parathion which have to be metabolized to their corresponding oxon forms to be effective. The overall model simulations indicated that additivity is obtained at oral lower dose levels of each chemical. At higher doses, antagonism by enzymatic competitive inhibition is the mode of interaction.

Pharmacokinetic and pharmacodynamic interaction for a binary mixture of chlorpyrifos and diazinon in the rat was examined in another study. It is reported that a dose-dependent inhibition of ChE was noted in tissues for both the single and coexposures and the overall relative potency for ChE inhibition was CPF/DZN > CPF > DZN (Timchalk *et al*., 2005).

The continuous and strong decrease in E_max_ value of ACh after incubating with ETF and/or CPF on rat ileum is possibly related to down-regulation of the number of receptors that ACh can be bound. Becerra *et al*. (2001) have examined the action of tacrine on muscarinic receptors in rat intestinal smooth muscle. The results indicate that tacrine has a biphasic effect: an increase of the ACh concentration because of it acting as an anticholinesterasic agent and a decrease on the contraction by ACh because of muscarinic receptor blockade. As the cholinesterase inhibitors prolong the maintenance of ACh in the bath, desensitisation occurs earlier with lower ACh concentrations. In addition, with these actions in vitro, long-term administration of cholinesterase inhibitors is known to result in a reduction in the number of muscarinic receptors and tacrine selectively down-regulates the number but not the affinity of M1 receptors. The results of the study have similar points with our study and the decrease in E_max_ value of ACh can be explained by down-regulation with ETF and/or CPF.

In conclusion, single and combined exposure to the agents used in this study caused agonistic interactions with regard to potency of ACh whereas they caused a decrease on E_max_ value of ACh. These findings suggest that exposure to these agents which have direct and indirect cholinergic effects, may cause developing clinical responses with small doses and earlier but the extent of toxicity will be lower.
